# The Use of Normothermic Machine Perfusion for Staged Combined Heart–Liver Transplant

**DOI:** 10.1155/crit/2288670

**Published:** 2025-06-23

**Authors:** Ye In Christopher Kwon, Aamir Khan, David A. Bruno, Zubair A. Hashmi, Josue Chery

**Affiliations:** ^1^Division of Cardiothoracic Surgery, Department of Surgery, Pauley Heart Center, Virginia Commonwealth University School of Medicine, Richmond, Virginia, USA; ^2^Division of Abdominal Transplant Surgery, Department of Surgery, Hume-Lee Transplant Center, Virginia Commonwealth University School of Medicine, Richmond, Virginia, USA

**Keywords:** cardiac cirrhosis, combined heart-liver transplant, heart failure, normothermic machine perfusion

## Abstract

**Introduction:** For patients with cardiac cirrhosis, combined heart–liver transplant (CHLT) has been increasingly performed with improving outcomes. The standard heart-then-liver approach may increase ischemic times and postreperfusion syndrome (PRS) risk. Achieving adequate hemodynamic stability may also pose a challenge. To mitigate these risks, we assessed the use of liver normothermic machine perfusion (NMP) in a staged CHLT.

**Case Presentation:** A 63-year-old male patient with diabetes, coronary artery disease, and NYHA Class III systolic heart failure presented to our center in cardiogenic shock. Subsequent liver biopsy found end-stage cirrhosis. He was bridged with an Impella 5.5 until a dual heart–liver donor became available. A standard heart transplant via redo sternotomy was performed on cardiopulmonary bypass (CPB). The chest was packed but left open in anticipation of the liver transplant. The liver was placed on NMP using the Organ Care System (TransMedics) with hepatic arterial and portal venous flows set at 350 and 0.8 mL/min, respectively. He received a staged liver transplant using the standard ‘piggyback' technique, 8 h after the heart transplant. There was minimal PRS and bleeding. Total time on NMP was 16.4 h. The chest and abdomen were closed at the end of the liver transplant. The postoperative course was complicated by acute renal failure requiring temporary hemodialysis. He was eventually discharged home, is now off dialysis, and continues to do well.

**Summary:** The NMP keeps the liver in an active metabolic state, allowing us to transplant the heart and establish optimal hemostasis to decrease blood product transfusion. This also allows time for proper postoperative fluid resuscitation and lactic acidosis clearance and helps achieve better hemodynamic stability with decreased inotrope/vasopressor doses. Additionally, the liver NMP is effective in minimizing complications related to PRS. A staged approach to CHLT using the NMP should be considered in such high-risk patients.

## 1. Introduction

Since its introduction by Starzl et al. in 1984 [1, 2], combined heart–liver transplant (CHLT) has been increasingly performed with improving outcomes for patients with concurrent end-stage heart and liver failure [3]. The incidence of liver cirrhosis in acute or chronic heart failure patients varies from 15% to 50% [4, 5]. However, advanced hepatic disease and cirrhosis are deemed contraindications for isolated heart transplantation due to the high short-term mortality rate [6, 7]. Thus, CHLT represents the only viable procedure for patients with extensive cardiac cirrhosis [8].

While various operative techniques for CHLTs have been described, the most common approach involves first completing the heart transplant on cardiopulmonary bypass (CPB), leaving the chest open, and performing the liver transplant on selective venovenous bypass (VVB) [9]. However, this standard method may be associated with increased cold and warm ischemic times and postreperfusion syndrome (PRS) risk [10–12]. Achieving adequate hemodynamic stability between the heart and the liver transplant may also present challenges, given the limited time the donor liver can tolerate on 4°C static cold storage before undergoing rapid, progressive organ deterioration [13]. To mitigate these effects, *ex situ* normothermic machine perfusion (NMP) of the liver allograft on the Organ Care System (TransMedics) has demonstrated promising outcomes compared to conventional ice storage, including reduction of early allograft dysfunction and ischemic biliary complications [14]. In donation after circulatory death (DCD) liver transplants, NMP has demonstrated adequate 1-year graft survival and ischemic cholangiopathy rates despite prolonged cold ischemic time prior to its initiation and higher early allograft dysfunction rates [15]. The advantages of NMP also involves reducing operative time, duration of hospital stay, rates of PRS, blood loss, and blood product transfusion [16]. Ultimately, it is critical to recognize that NMP may help increase the donor organ pool by allowing for organs to travel longer distances [17]. This report describes our experience utilizing the liver NMP in a staged CHLT with acceptable patient outcomes. The patient provided written informed consent to report his case details and imaging studies. This manuscript was presented at the 45^th^ International Society for Heart and Lung Transplantation Annual Meeting & Scientific Sessions on April 27–30, 2025 in Boston, MA, United States [18].

## 2. Case Presentation

### 2.1. History and Presentation

**Figure 1:**
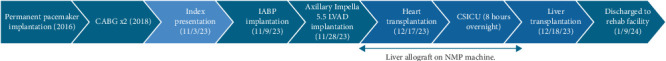
A schematic of the patient's surgical history and index case progression. Abbreviations: CABG: coronary artery bypass graft; CSICU: cardiac surgery intensive care unit; IABP: intra-aortic balloon pump; LVAD: left ventricular assist device; NMP: normothermic machine perfusion.

The patient is a 63-year-old male in cardiogenic shock who presented to the Virginia Commonwealth University Health Medical Center in December 2023. His past medical history included Diabetes Mellitus Type 2, coronary artery disease requiring a vessel coronary artery bypass graft, and New York Heart Association Class 3 chronic systolic heart failure (Figure 1). On admission, he had frequent premature ventricular contractions and multiple runs of nonsustained ventricular tachycardia. His arrhythmia precluded further escalation of inotropic support. While the patient was being evaluated for heart transplantation by our multidisciplinary team, an intra-aortic balloon pump was placed for mechanical support, which remained for 19 days. However, he started to become deconditioned, and thus, a temporary left ventricular assist device, Impella 5.5 (Abiomed), was inserted via his right axillary artery for continued circulatory support.

### 2.2. Heart Failure Evaluation

**Figure 2:**
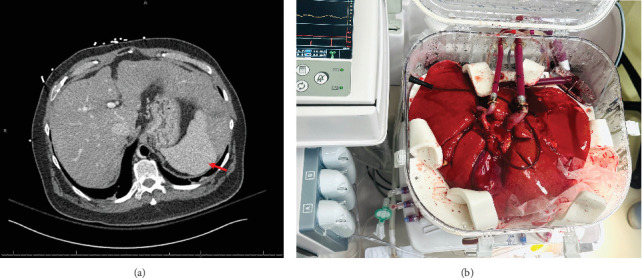
Preoperative liver assessment and intraoperative donor liver allograft preservation. (a) Preoperative axial contrast-enhanced computed tomography image demonstrating 1.3 × 2.9 cm hypoattenuating lesion of the left lobe of the liver (red arrow). (b) Normothermic machine perfusion of the donor liver on the Organ Care System (TransMedics).

Initial evaluation with a transthoracic echocardiogram revealed a left ventricular ejection fraction (LVEF) of 10% with moderate–severe hypokinesis and dilatation of both ventricles. The systolic function of the right ventricle was moderate to severely reduced. The right heart catheterization demonstrated a mean right atrial pressure of 20, pulmonary capillary wedge pressure of 22, mean pulmonary arterial pressure of 32, and a cardiac index of 1.63 L/min/m^2^ while on 5 mic/kg/min of dobutamine. His left heart catheterization demonstrated progressive three-vessel coronary artery disease with a patent reverse saphenous graft to a disease obtuse marginal branch and a patent yet atretic left internal mammary graft to a diseased and small left anterior descending artery. No further revascularizations were realistic. Additional contrast-enhanced computed tomography evaluation demonstrated nodular liver morphology suggesting cirrhosis (Figure 2a). Subsequently, a liver biopsy confirmed the presence of end-stage hepatic parenchyma cirrhosis and mild macrovesicular steatosis with elevated portal pressures and a portosystemic gradient of 9 mmHg. His model for end-stage liver disease (MELD) 3.0 score was 22. In the absence of extensive alcohol use, the etiology of his cirrhosis was deemed to be secondary to his cardiomyopathy or metabolic dysfunction-associated steatohepatitis. All further routine workups were within normal limits. The consensus from both the heart failure team and the abdominal transplant team was to list the patient for both a heart and liver transplantation. Given his cirrhosis, a durable LVAD was not a viable option.

### 2.3. CHLT

A suitable dual heart–liver donor without any high-risk features was identified for him, 19 days after the placement of Impella 5.5. We proceeded with a redo sternotomy, extensive lysis of adhesions, explanation of the Impella 5.5, and bicaval orthotopic heart transplantation. Total CPB time was 308 min, and total ischemic time on the newly transplanted heart was 183 min with 77 min of warm ischemic time. The patient received two units of packed red blood cells and one unit of platelets intraoperatively. The patient was weaned off CPB without any difficulty. The heart was decannulated, and protamine was administered. Intraoperative echocardiogram demonstrated LVEF of 55%–60% with no right ventricular dysfunction. In anticipation of the subsequent liver transplant, the patient's chest was left open. The sternal edges were packed, and the surgical wound was closed with a plastic membrane sewn to the skin. The occlusive dressing, Ioban, was placed on the chest.

While the heart transplant was being performed, the liver was placed on the NMP (Figure 2b). On NMP, the hepatic arterial and portal venous flows were set at 350 and 0.8 mL/min, respectively. The patient returned to the operating room for a staged liver transplant using the standard ‘piggyback' technique, 8 h following the conclusion of the heart transplant. The chest was reopened, and the sternal retractor was applied while the abdominal team opened. A primed CPB machine was brought to the room, and all lines were passed as routinely for a pump case. Purse string sutures were placed on the distal ascending aorta and the right atrial appendage. These were done so that we could proceed with emergent CPB should the patient become unstable. The abdominal transplant team then proceeded with a suprahepatic vena caval anastomosis, which was performed by running 3-0 Prolene sutures. The portal vein anastomosis was performed by running 6-0 Prolene sutures. The suprahepatic vena caval clamp was removed, followed by the portal venous clamp, with minimal reperfusion syndrome and bleeding. Hepatic arterial anastomoses were performed using 6-0 Prolene sutures, demonstrating vigorous blood flow. Finally, the bile duct reconstruction was performed in a ‘duct-to-duct' fashion with running 6-0 Polydioxanone sutures. The total time on NMP was 16.4 h. The liver experienced 127 min of cold ischemia time and 30 min of warm ischemia time. Upon completion of the liver transplantation, the chest was washed out and closed in a standard fashion. The abdomen was also closed in the standard fashion.

### 2.4. Postoperative Course and Follow-Up

His postoperative course was complicated by Stage 3 acute kidney injury, requiring continuous renal replacement therapy. On postoperative Day 27, he was discharged to a rehabilitation facility requiring intermittent hemodialysis and standard immunosuppression. At discharge, his liver function tests showed aspartate aminotransferase of 15 U/L (normal range 0–50 U/L), alanine aminotransferase of 18 U/L (normal range 0–60 U/L), alkaline phosphatase of 106 U/L (normal range 40–120 U/L), and total bilirubin of 0.5 mg/dL (0–1.3 mg/dL). The last follow-up was conducted in May 2025. Approximately 16 months after CHLT, the patient continues to do well with LVEF of 55%–60%; has MELD 3.0 score of 10, no residual neurologic deficits, and stable internal jugular venous thrombosis; and is off hemodialysis.

## 3. Discussion

CHLT is a complex yet potentially life-saving procedure requiring a highly experienced, specialized, and multidisciplinary team. Generally, patients exhibiting three or more clinical manifestations of portal hypertension, or those presenting any indication of portal hypertension alongside F3 or more severe fibrosis as evidenced by liver biopsy, are preferentially considered for CHLT rather than isolated heart transplantation [19]. These determinants, in conjunction with diagnostic imaging and hepatic function assessments, serve to determine which patients are least likely to sustain the success of isolated heart transplantation due to hepatic insufficiency. While the standard sequential heart-then-liver approach is the most common technique for CHLT [9], the staged approach offers several key advantages compared to the standard heart-then-liver approach.

First, the NMP preserves and keeps the donor liver in a metabolically active, nonischemic state by perfusing the donor organ with warm, oxygenated, nutrient-dense, blood-based perfusate [14]. This allows us to space out the heart and the liver transplant, thereby helping the patient to adequately circumvent the immediate postoperative hemodynamic instability after the heart transplant. The high-risk profiles of patients undergoing CHLT pose unique perioperative challenges, which require meticulous details regarding transfusion and managing coagulopathy. A recent study with *en bloc* CHLT reported requiring, on average, 24 units of packed red blood cells intraoperatively [19]. In pediatric CHLT, many patients with preoperative decompensated heart and liver failure require massive blood transfusions post-CPB, ranging from one to three times the patient's estimated blood volume [20, 21]. However, in our case, we not only necessitated far less intraoperative transfusion but also needed no further blood transfusion during the additional postheart transplant standby time. Nevertheless, CHLT is a much more extensive surgery than isolated or liver heart transplantation and thus should be reserved for a highly selective group of patients.

Second, the staged approach may be beneficial in establishing proper postoperative fluid resuscitation and clearance of lactic acidosis. Importantly, high inotrope scores postheart transplant have been previously associated with an increased risk of mortality [22]. Our approach allows us to adequately decrease inotrope and vasopressor doses postheart transplant. In this case, our patient remained on 0.05 mcg/kg/min of epinephrine and 3 mcg/kg/min isoproterenol postheart transplant. After the liver transplant, we were appropriately able to further decrease epinephrine to 0.01 mcg/kg/min and isoproterenol to 2 mcg/kg/min while preserving and optimizing ventricular function. Another critical yet poorly studied aspect of the staged approach is the delayed sternal and abdominal closure, which could provide extra space for edematous allografts [19]. In our case, this has been a valuable technique for the postoperative management of patients who may require additional CPB or mechanical circulatory support during the liver transplant. A multidisciplinary perfusion team can be an invaluable resource in such scenarios.

The early CHLT techniques described by Shaw et al. employed both CPB and portal vein decompression via portal drainage during the liver transplant [2]. However, posttransplant coagulopathy, brought on by the prolonged use of heparin, presented significant challenges to this approach, requiring several hours for resolution [2]. Although it is no longer the standard practice to complete CHLT all under CPB, some argue that the risks associated with prolonged CPB may be outweighed by the reduced metabolic dysfunction with hepatic reperfusion, which could safeguard the heart allograft from complications such as hyperkalemia, fluid overload, and acidosis [23, 24]. It is also often described that a combined procedure on CPB may reduce liver cold ischemic time by eliminating the time required to reverse anticoagulation [23, 24]. However, NMP for the donor liver addresses these issues without requiring CPB. Compared to the 348 min reported by Rauchfuss et al. when completing CHLT entirely on CPB, our experience with the liver allograft on NMP has halved that cold ischemic time. The major alternative to performing CHLT on CPB is using the ‘piggyback' technique with partial inferior vena cava clamping or VVB [25]. We demonstrate that the newly implanted heart can effectively tolerate the caval clamping during the ‘piggyback' technique without needing VVB. It is plausible that the reduced anhepatic phase and total operating time during the liver transplant may help increase renal flow and venous return to the heart, thereby further improving hemodynamic stability [25, 26].

Third, the liver NMP has been effective in minimizing complications related to PRS, an often dreaded complication of liver transplant representing a significant risk factor for recipient mortality and graft failure [10]. The initial PROTECT trial demonstrated that compared to static cold storage, NMP significantly reduces the risk of PRS [14]. While the underlying mechanism of this effect is not well understood, it is possible that NMP, by replenishing the metabolic status of the donor liver, minimizes the initial ischemic insult, thereby reducing subsequent RPS [12, 27]. Our case also serves to demonstrate the upper limits of liver allograft preservation time on NMP, which extended beyond 16 h compared to 5.52 to 11 h demonstrated by recent reports of similar techniques [28, 29]. Thus, we further add to the growing body of evidence demonstrating the feasibility and safety of accepting donor livers from a further distance in CHLT.

Fourth, another advantage of the NMP system is the ability to assess liver hemodynamics and function throughout the preservation period continuously [14]. The utility and viability of NMP have also been demonstrated in several high-risk clinical situations, including in discarded livers [30], retransplants [31], and livers from DCD [32]. These scenarios may help to address some of the main challenges in finding an adequate pair of organs in CHLT. In instances when the liver is deemed suboptimal for transplantation, NMP preservation may also allow for reallocation of the liver to another suitable candidate.

Finally, an important aspect of this case's clinical outcomes is the freedom from moderate-to-severe graft rejection and chronic rejection. While the exact mechanism remains unclear, it has been hypothesized that the liver allograft has some immunoprotective properties to the heart allograft [33, 34]. Our center's immunosuppression regimen for CHLT does not significantly deviate from that of isolated heart transplants. However, decreasing the amount of immunosuppressive therapies in patients who undergo CHLT [19] may be possible. Our patient continues to do well and is being actively managed by a multidisciplinary cardiology and hepatology team to reduce the risk of rejection.

## 4. Conclusions

While staged CHLT is typically reserved for scenarios where the heart and the liver are procured from different donors [9, 35], we believe that select high-risk patients with cardiac cirrhosis may benefit from staged CHLT using the liver NMP system. We demonstrate the longest donor liver preservation time on the NMP system in the setting of staged CHLT with excellent dual graft function and long-term recipient outcomes. The key advantages of this strategy include establishing optimal hemostasis after the initial heart transplant and minimizing complications related to PRS after the liver transplant. Without a consensus regarding the optimal surgical technique for CHLT, careful donor and recipient selection and a multidisciplinary team approach to surgical planning are paramount to maximize patient and multigraft survival benefits.

## Data Availability

The data that support the findings of this study are available from the corresponding author upon reasonable request.

## Nomenclature

CHLT: combined heart–liver transplant
CPB: cardiopulmonary bypass
HT: heart transplant
LT: liver transplant
LVAD: left ventricular assist device
LVEF: left ventricular ejection fraction
NMP: normothermic machine perfusion
NYHA: New York Heart Association
MELD: model for end-stage liver disease
PRS: postreperfusion syndrome
VVB: venovenous bypass
